# Reproduction of Characteristics of Extracellular Matrices in Specific Longitudinal Depth Zone Cartilage within Spherical Organoids in Response to Changes in Osmotic Pressure

**DOI:** 10.3390/ijms19051507

**Published:** 2018-05-18

**Authors:** Eiichiro Takada, Shuichi Mizuno

**Affiliations:** Department of Orthopedic Surgery, Brigham and Women’s Hospital, Harvard Medical School, Boston, MA 02115, USA; eiichiro.takada.hy@hitachi-hightech.com

**Keywords:** molecular profile, extracellular matrix, osmotic pressure, depth articular cartilage, spheroidal organoid, cartilage regeneration

## Abstract

Articular cartilage is compressed with joint-loading and weight-bearing stresses, followed by a bulging of the tissue during times of off-loading. This loading and off-loading causes changes in water content, and thus alterations in osmotic pressure. Another unique characteristic of articular cartilage is that it has longitudinal depth: surface, middle, and deep zones. Since each zone is composed of unique components of highly negative extracellular matrices, each zone has a different level of osmotic pressure. It was unclear how changes in osmotic pressure affected chondrocyte matrix turnover in specific longitudinal zones. Therefore, we hypothesized that a change in extrinsic osmotic pressure would alter the production of extracellular matrices by zone-specific chondrocytes. We incubated spheroidal cartilage organoids, formed by specific longitudinal depth zone-derived chondrocytes, under different levels of osmotic pressure. We compared the gene expression and the immunohistology of the matrix proteins produced by the zone-specific chondrocytes. We found that high osmotic pressure significantly upregulated the transient expression of aggrecan and collagen type-II by all zone-derived chondrocytes (*p* < 0.05). At a high osmotic pressure, surface-zone chondrocytes significantly upregulated the expression of collagen type-I (*p* < 0.05), and middle- and deep-zone chondrocytes significantly upregulated matrix metalloproteinase-13 (*p* < 0.05). The spheroids, once exposed to high osmotic pressure, accumulated extracellular matrices with empty spaces. Our findings show that chondrocytes have zone-specific turnover of extracellular matrices in response to changes in osmotic pressure.

## 1. Introduction

Articular cartilage contains an abundant amount of negatively charged sulfated glycosaminoglycan (e.g., chondroitin sulfate) in the extracellular matrix (ECM), resulting in significantly higher osmotic pressure (OP) than in other tissues [1,2]. Because of weight bearing and joint loading, articular cartilage exudes interstitial water into synovium space and suchondral bone, but absorbs the water from these tissues after the loadings [3]. This in- and outward-moving interstitial water has the potential to alter the volume of the cartilage tissue and ultimately to trigger changes in OP (ΔOP) [4]. The damage to articular cartilage causes a loss of ECM macromolecules and results in ΔOP and osteoarthritis (OA) development over time [5].

Articular cartilage has histologically distinct longitudinal depth zones; surface zone (SZ), middle zone (MZ), and deep zone (DZ), resulting in unique ECM components [1,3]. Each zone consists of various concentrations of collagen types I (COL-1) and II (COL-2) and aggrecan. Typically, a thin layer of SZ accumulates a relatively large amount of collagen type I when compared with other zones. Collagen type II and aggrecan are produced by all zones, particularly MZ. Aggrecan has an abundant negative fixed charge density, which causes relatively higher local osmotic pressure when compared with SZ and DZ. DZ includes calcification with dense COL-1 next to a subchondral bone.

It was unclear how ΔOP had the potential to alter metabolic functions in each zone. Thus, we hypothesized that the ΔOP alters cell origin-dependent metabolic functions by specific depth zone-derived articular chondrocytes. Recently, the effects of OP on chondrocyte metabolism have been studied using a chondrocyte suspension [6], a monolayer [7], a cell construct [8], and a cartilage explant [9] at defined OP in culture medium. However, these experimental models were limited in their ability to reproduce longitudinal depth. To test our hypothesis, we used a self-assembled spherical chondrocyte organoid, a so-called “spheroid”. This spheroid demonstrated a unique gene expression profile and histological characteristics by chondrocytes isolated from the specific depth zones in articular cartilage [10]. The specific zone-derived chondrocytes formed a spherical cell aggregation and accumulated newly synthesized ECM having negatively charged fixed-charge density. Thus, this spheroid model allowed for the difference of OP between the inside (accumulated ECM) and outside (culture medium) of the spheroid, which has the potential to mimic the difference of OP between cartilage tissue and synovial fluid. Although a chondrocyte pellet formed with centrifugation has been used for investigating cell differentiation and metabolic function, a symmetrical spheroid model has the advantage of reproducing zone-specific characteristics of chondrocytes [10,11]. In this study, we incubated spheroids with ΔOP by switching two culture mediums having different OPs. We then measured RNA expressions of the following ECM components: anabolic molecules; aggrecan core protein *(Agg-core*); *Col-1*; *Col-2*; and a degenerative molecule, matrix metalloproteinase (*MMP-13*), by each zone-derived chondrocyte at days 10, 13, and 21, and determined the localization of these corresponding molecules immunohistologically [12,13,14].

## 2. Results

**Figure 1:**
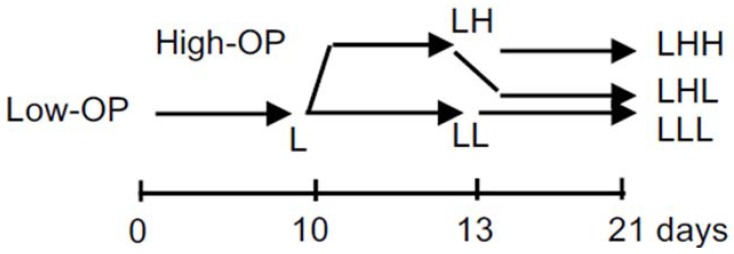
Algorithm of changes in osmotic pressure of culture medium. Osmotic pressure of culture medium was changed or maintained at days 10 and 13. L indicates low-OP; LL and LH indicate continuously low-OP and changed OP from low to high at day 10, respectively; LLL, LHL, and LHH indicate continuously low-OP for 21 days, changed OP from low to high at day 10 followed by changed OP from high to low at day 13, and changed OP from low to high at day 10 followed by continuously high-OP, respectively.

We incubated chondrocytes at Low-OP (320 mOsm) from 0 to day 10 (L), and changed the medium to either continuously Low-OP (LL) or High-OP (450 mOsm, LH) (Figure 1). Then, we changed the medium to either continuously Low-OP (LLL) or continuously High-OP (LHH), or switched it back from High-OP to Low-OP (LHL) at day 13, and incubated the spheroids for another six days before day 21. Spheroids were collected for molecular evaluation at days 10, 13, and 21, when the culture medium was changed, as well as for immunohistological evaluation at day 21. We conducted the experiments with the same algorithm of changing in OP four times.

### 2.1. Formation of Spheroids

**Figure 2:**
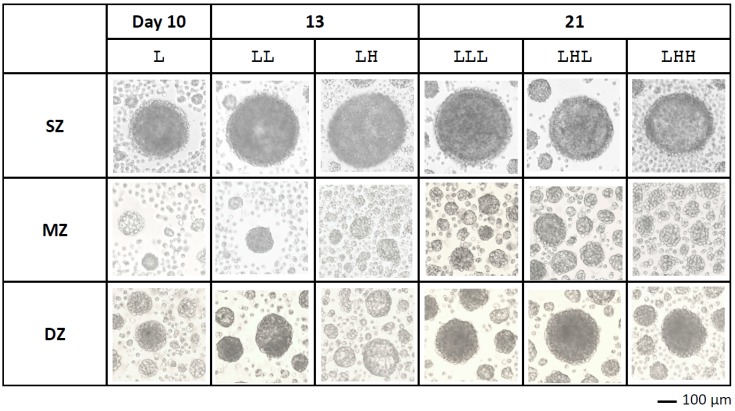
Photomicrographs of spheroids observed with an inverted microscope at days 10, 13, and 21. SZ: Surface zone, MZ: Middle zone, DZ: Deep zone. A bar indicates 100 µm.

Chondrocytes isolated from SZ, MZ, and DZ in articular cartilage settled on a non-adherent well by three to four hours after cell seeding. After four days of the culture, the chondrocytes aggregated, and subsequently formed spheroids by day 7 (Figure 2). Particularly, SZ cells formed one spheroid (>100 µm) in each well by day 4. On the other hand, MZ cells aggregated with multiple cells by day 4, and subsequently formed several smaller spheroids (~50 µm in diameter) by day 7. DZ cells showed slightly different characteristics from other zone-derived cells. DZ cells formed one relatively large spheroid and several smaller ones surrounding the larger one like satellites. DZ spheroids at day 21 were larger than those at day 10.

### 2.2. Gene Expression Profiles

**Figure 3:**
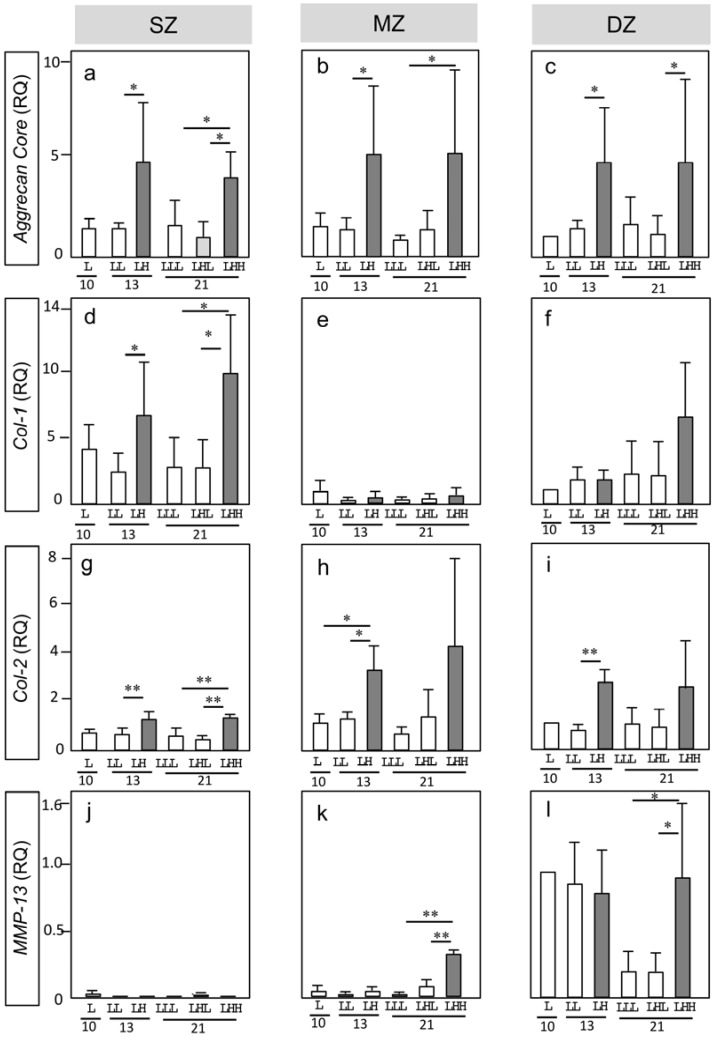
Gene expression by longitudinal depth zone-derived chondrocytes forming spheroids. Relative quantity (RQ) Asterisks indicate significant difference among groups (* *p* < 0.05, ** *p* < 0.01). A bar indicates SD. Collagen type Iα1 (*Col-1*); collagen type IIα1 (*Col-2*); matrix metalloproteinase-13 (*MMP-13*).

We measured RNA expressions of the following ECM components: *Agg-core*, *Col-1*, and *Col-2*, and *MMP-13,* by each zone-derived chondrocyte at days 10, 13, and 21 (Figure 3). The relative quantity (RQ) of each gene expression was compared to that of DZ at day 10, whose value was defined as 1.0.

*Agg*: We measured the gene expression of *Agg-core* as a part of the aggrecan produced by chondrocytes (Figure 3a–c). At Low-OP, SZ, MZ, and DZ expressed *Agg-core* at levels similar to the DZ control at day 4 throughout the experiments. By descending OP, chondrocytes from all zones upregulated *Agg-core* significantly; namely, two to three times greater than at Low-OP (LL) at day 10. By switching the High-OP back to Low-OP at day 13 (LHL), the expressions of *Agg-core* by chondrocytes from all zones returned to the level of the Low-OP (LLL). At High-OP (LHH), however, chondrocytes from all zones maintained significant similar upregulation levels of *Agg-core* from days 13 to 21.

*Col-1*: We measured the gene expression of *Col-1* to evaluate anabolic activity related to the production of ECM in articular cartilage (Figure 3d–f). SZ at Low-OP upregulated *Col-1* three times more than the DZ control at days 10 and 21. By ascending OP at day 13, SZ upregulated *Col-1* significantly three times more than that at Low-OP. On the other hand, MZ expressed *Col-1* at a level similar to the DZ control at Low-OP by day 10. By ascending OP, MZ expressed *Col-1* at a negligibly lower level when compared with the DZ control by day 21. DZ maintained the *Col-1* expression at a similar level throughout the experiments at Low-OP, except for High-OP at day 21. DZ at High-OP upregulated *Col-1* significantly; namely, three times more than it did at Low-OP. Overall, the measurement of *Col-1* expression demonstrates that SZ and DZ responded sensitively to High-OP.

*Col-2*: We measured the gene expression of *Col-2* to evaluate anabolic activity related to the production of typical cartilage ECM (Figure 3g–i). SZ and MZ at Low-OP expressed *Col-2* at a similar or lower level when compared with the DZ control throughout the experiments. However, by ascending OP, MZ significantly upregulated *Col-2* 2.5 and 4 times more than Low-OP at days 13 and 21, respectively. DZ at Low-OP expressed *Col-2* at a level similar to the control throughout the experiments. However, by ascending OP, DZ significantly upregulated *Col-2* 3 and 2.5 times more than the control at days 13 and 21, respectively. Overall, the measurement of *Col-2* expression demonstrates that MZ and DZ responded sensitively to High-OP.

*Mmp-13*: We measured the gene expression of *Mmp-13* to evaluate degenerative activity by specific zone-derived chondrocytes (Figure 3j–l). SZ and MZ expressed *Mmp-13* at a negligibly lower level when compared with the DZ control, except for MZ at High-OP by day 21. On the other hand, DZ declined *Mmp-13* significantly one-fifth at Low-OP by day 21.

### 2.3. Histological Characteristics of Spheroids

**Figure 4:**
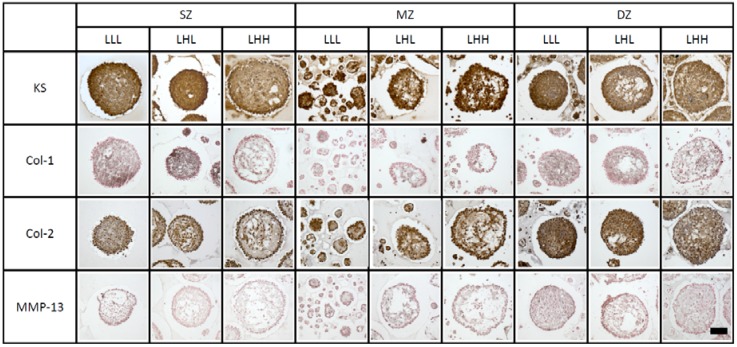
Immunohistochemistry of spheroids formed with bovine longitudinal depth zone-derived chondrocytes at day 21. Keratan sulfate: KS, collagen type-II: Col-2, collagen type-I: Col-1, and matrix metalloproteinase-13: MMP-13. Brown stain is positive, and brown/dark blue indicates counterstaining of nuclei. Black deposits indicate COL-1, and red indicates counterstaining of nuclei. A bar indicates 100 µm.

At day 21, we consolidated the spheroids from 24 wells of each OP condition (LLL, LHL, and LHH) into one sample and embedded them in 30 µL of 1.5% agarose for efficient sectioning. This agarose remained in the section so that the debris and ECM secreted by the cells were also stained with the antibodies. Ten-micrometer cross sections of the spheroids were stained with antibodies against keratan sulfate (KS), COL-1, COL-2, and MMP-13 (Figure 4).

KS and nuclei appeared in brown and dark blue, respectively. Aggrecan is composed of a specific, but minor amount of KS so that *Agg-core* and KS are supposed to be consistent. SZ cells accumulated abundant KS within spheroids at both OPs. Empty space and light brown deposits were seen among cells within a spheroid at LHH. Abundant KS was accumulated inside MZ spheroids, and particularly denser KS in the outmost spheroid’s layer, at both OPs. With a treatment of transitional (LHL) or continuously High-OP (LHH), the MZ spheroids had empty spaces. DZ cells accumulated abundant KS within spheroids at Low-OP medium (LLL). With a treatment of transitional (LHL) or continuously High-OP (LHH), the DZ spheroids accumulated denser KS in the outmost layer of the spheroid and had empty spaces. Overall, empty spaces were seen in spheroids formed by SZ, MZ, and DZ cells at High-OP (LHL, LHH).

COL-1 and nuclei appeared as fine black fibers and in red, respectively. Although COL-1 was not cartilage-specific ECM, it was found in the surface layer of articular cartilage. SZ cells accumulated COL-1 at Low-OP (LLL). With a treatment of transitional High-OP (LHL), the spheroids accumulated denser COL-1 in the outmost layer of the spheroid. Less accumulation was seen at the center and the outmost layer of the spheroids, but more fibrous COL-1 was seen at High-OP (LHH). MZ cells accumulated much less COL-1 within spheroids when compared with other zones. DZ cells accumulated abundant COL-1 at Low-OP (LLL). Denser COL-1 was seen between the center and outer cells in a concentric fashion. With a treatment of transitional High-OP (LHL), fibrous COL-1 and empty spaces were seen among cells.

COL-2 and nuclei appeared in brown and dark blue, respectively. SZ cells accumulated COL-2 around cells at Low-OP (LLL). With a treatment of transitional High-OP (LHL), the spheroid had empty spaces and denser COL-2 in the outmost layer of the spheroid (LHL). The SZ cells accumulated COL-2 between the center and outmost layer of the spheroid at continuously High-OP (LHH). MZ cells accumulated COL-2 in the outmostlayer of the spheroid at any OP and had non-stained empty spaces at High-OP. DZ cells accumulated COL-2 inside and in denser quantities in the outmost cellular layer at Low-OP.

MMP-13 and nuclei appeared as fine black deposits and in red, respectively. Particularly, DZ cells accumulated denser MMP-13 when compared with other zones. Notable differences were not seen between different OPs.

## 3. Discussion

### 3.1. Morphological Characteristics of Spheroids Formed with Specific Longitudinal Depth Zone-Derived Chondrocytes in Response to ΔOP

We previously demonstrated that chondrocytes isolated from a specific longitudinal depth zone in articular cartilage formed a spheroid expressing unique characteristics of the zone [10]. The average size and number of the spheroids formed with the same number of seeded cells depended on the zone. In this study, we used this spheroid model to clarify gene and protein expression in specific depth zones of articular cartilage in response to ΔOP.

A chondrocyte spheroid allowed for the accumulation of newly synthesized ECM within the spherical compartment formed with an outer cellular layer. This three-dimensional configuration allowed us to investigate the effects of ΔOP by alternating different OP culture mediums. We expected that the outer cellular layer would mimic the compartment of a semipermeable membrane pouch, because the cells were connecting seamlessly to reproduce the Gibbs–Donnan effect.

We thought that a High-OP in medium with a high concentration of sodium chloride (450 mOsm) would make a spheroid shrink [15]. In this study, however, the spheroids swelled at High-OP. This difference may have caused ECM accumulation within the spheroid due to the high fixed negative charge of the ECM [2]. Thus, we speculated that this ECM had the potential to attract positively charged ions (solute) and result in High-OP within the ECM, followed by the absorption of water, to be equivalent to the OP in medium. In addition, we speculated that the swelling and shrinkage of the spheroid would mimic interstitial OP within articular cartilage tissue. This spheroidal shape would also be useful to evaluate the gradient of ECM compared to a slab or a cylindrical shape, because the configuration of the spheroid was point-symmetric [16]. Thus, the diffusion within the spheroid occurred uniformly, which makes physicochemical events easy to understand.

### 3.2. Metabolic Functions of Chondrocytes Isolated from a Specific Zone in Response to ΔOP

We hypothesized that chondrocytes isolated from a specific longitudinal depth zone would express unique characteristics to maintain ECM in response to ΔOP, either ascending or descending OP. Each zone is composed of unique ECM components, which directly impact cell behavior and shape [3]. Thus, we measured gene expressions of typical ECM molecules: *Agg*, *Col-1*, *Col-2*, and *Mmp-13,* and stained these molecules with corresponding antibodies. We chose *Agg-core*, *Col-2*, and KS, typically seen in MZ; *Col-1* and COL-1, typically seen in SZ and DZ; and *Mmp-13* and MMP-13, which is related to ECM turnover.

We changed OP by replacing the first 10 days’ culture medium at 320 mOsm with the second one at 450 mOsm, and with supplemental sodium chloride at days 10 and 13. Although other solutes (e.g., mannitol) could be used to change OP, we chose sodium chloride—which we used in our previous studies—and others for comparison [6,9,17]. Normal OP in native articular cartilage was higher than the physiologically equivalent OP, known as saline. Because the daily loading cycle on the knee was supposed to be intermittent, we sought to compare a state of constant OP and ΔOP, with the exception of osmotic shock (a momentary change in OP). Thus, we examined ΔOP in two phases, including acclimatization to the constant OP and changes in either ascending or descending OP by switching two different mediums. The duration of the shortest exposure to OP was set at three days, in order to detect gene expression levels per our other studies [17].

SZ-derived chondrocytes: Our subordinate hypothesis was that SZ cells would be non-reactive in response to ΔOP. Since SZ in native cartilage is composed of thin fibrous ECM (e.g., COL-1) and less negatively charged ECM to withstand compressive stress and wear, we thought ΔOP would not impact the SZ. In this study, SZ cells showed greater upregulation of *Col-1* at High-OP compared with Low-OP and with other zones, and almost no upregulation of *Mmp-13* at any OP level. Thus, these results did not support our hypothesis. Distinct differences between SZ cells and the adjacent MZ cells were seen in *Col-1* and *Col-2* expressions. Since histologically looser ECM was seen within a spheroid, it seems that the spheroid expanded and absorbed water. Therefore, we thought that the upregulation of *Col-1* was preventing disruption to its spherical shape due to an expansion [18,19]. Similarly, in the surface of native cartilage, SZ had the roles of withstanding joint loading and weight bearing, as well as maintaining ECM within the tissue. Interestingly, upregulation of *Mmp-13* by SZ cells was not seen. We speculated that SZ exposed to catabolic enzymes produced other joint tissues (e.g., synovium membrane) [20].

MZ-derived chondrocytes: Our subordinate hypothesis was that MZ cells would be sensitive in response to ΔOP. MZ is composed of highly negatively charged aggrecan, partially stained with anti-KS, which has the ability to absorb interstitial water, resulting in ΔOP. MZ cells upregulated anabolic genes and created distinct empty spaces within a spheroid at High-OP when compared with Low-OP. Compared with other zone cells, the MZ spheroid had an irregularly round shape. *Col-1* expression was far less than those of SZ and DZ. Thus, less COL-1 likely caused limited tension to form a round shape. In native cartilage, another role of SZ is to cover and protect MZ. When damaged cartilage does not have SZ, MZ could be exposed to synovial fluid. Anti-KS heavily stained MZ spheroids and the area around them. Thus, the MZ spheroid reproduced the swelling of MZ in native cartilage. We speculated that MZ cells would have the ability to produce a substantial amount of chondroitin sulfate and secrete it into the medium and within the spheroids at any OP level ^22^. This characteristic is one of reasons that explain lower cell density in MZ and abundant ECM accumulation. In addition, expression levels of *Agg-core* and *Col-2* at LHL showed a higher trend than those at LLL. Our hypothesis had to change to reflect that ΔOP moderately impacts production and accumulation of KS and COL-2 by MZ cells. Thus, in native cartilage, we think that MZ allows for the maintenance of ECM against ΔOP.

DZ-derived chondrocytes: Our subordinate hypothesis was that DZ cells would stimulate aggrecan and COL-1 production in response to ΔOP. We thought that DZ cells had the potential to stimulate aggrecan and COL-1 production in response to ΔOP, because DZ was composed of sulfated and calcified ECM, and was located next to a subchondral bone [21,22,23]. This hypothesis was supported by our results. Interestingly, DZ cells upregulated *Mmp-13* more than other zone cells by day 13 at any OP level, and sustained that upregulation at High-OP by day 21. Since upregulation of *Mmp-13* was seen at an early time when other zones did not show it, DZ cells resumed the activity of synthesis of MMP-13 at High-OP. We speculated that DZ cells had the potential to turn over ECM or remodel by themselves.

In conclusion and perspective, we clarified the effects of ΔOP on metabolic functions of chondrocytes isolated from specific longitudinal depth zones using their self-formed spheroids. Without multiple passages and monolayer culture, chondrocytes maintained metabolic functions to produce their typical ECMs and accumulated within and around a spheroid. ΔOP had the potential to impact various metabolic functions characterizing each distinctive zone [24]. This spheroid model will be useful to understand morphogenesis, homeostasis, and pathogenesis of longitudinal depth zones in articular cartilage in response to physicochemical stresses. For example, OA progresses from the surface to the deep zone in vivo. Thus, SZ spheroidal organoids can mimic early OA when the surface layer of the spheroid is damaged. ΔOP mimic daily on/off stresses through the surface layer. MZ spheroidal organoids can mimic mid-stage OA because of their direct exposure to culture medium. MZ cells surrounded by newly synthesized extracellular matrices have the potential to maintain their homeostasis in response to repeated changes in OP. In addition, DZ spherical organoids can mimic late-stage OA and calcification of cartilage. We will include stem cell behavior in SZ, small molecules in MZ (e.g., cartilage intermediate layer protein), and unique sets of cells (e.g., chondron) in MZ and DZ [25,26,27,28], in further studies.

## 4. Materials and Methods

We focused on the effects of changes in osmotic pressure on histogenesis and its related ECM production in distinct longitudinal depth zones of articular cartilage using this spherical organoid model. We incubated the spheroids in culture medium with a defined OP. Because OP in articular cartilage is altered by weight bearing, joint loading, and off-loading in daily cycles, we mimicked the ascending and descending changes in OP during the culture. We evaluated the gene expression and histology in chondrocytes isolated from distinct longitudinal depth zones in response to these changes in OP.

### 4.1. Cell Isolation from Specific Depth Zones in Articular Cartilage

We used chondrocytes isolated from specific longitudinal depth zones in bovine articular cartilage, as previously described [10,17,29]. Briefly, the forelimbs of two- to three-week-old calves were obtained from a local abattoir within three hours after slaughter (United States Department of Agriculture certified). We repeated this experiment four times for conducting statistical analysis. Under aseptic conditions, the cartilage pieces (<5 × 5 × 3–5 mm) were harvested from the forelimb distal chondyle. We removed specific cartilage zones (SZ, MZ, and DZ) by slicing the SZ (100–200 µm thick) from the surface of the cartilage and the DZ (200–300 µm thick) from the subchondral bone with #22 and 15 blades (BD, Franklin Lakes, NJ, USA). The remainder was defined as MZ. Collagenase CLS 1 (Worthington, Lakewood, NJ, USA) was dissolved at 0.15% in Ham’s F-12 medium (Life Technology, Grand Island, NY, USA) with 100 units/mL penicillin and 100 µg/mL streptomycin (Life Technology) and sterilized with a 0.45 µm filter (Nalgene™, Thermo Fisher Scientific, Rochester, NY, USA). The slices were minced and gently digested with the collagenase for twelve hours at 37 °C on a rotary shaker. The dispersed bovine articular chondrocytes (bACs) were sheaved with a 70-µm-cell strainer (Falcon^TM^, Fisher, Pittsburgh, PA, USA) to remove debris. The cells were rinsed with Dulbecco’s-phosphate buffer saline (D-PBS, Life Technology) twice and re-suspended in Dulbecco’s minimal Eagle medium (DMEM, Life Technology)/Ham’s F-12 medium (1:1) with 10% fetal bovine serum, 100 units/mL penicillin, and 100 µg/mL streptomycin.

We seeded one thousand bACs isolated from each zone in 150 µL of the medium into a well of a round-bottom, 96-well plate (Sumitomo, Tokyo, Japan), and incubated them at 5% CO_2_ in air and at 37 °C for ten days. One hundred microliters of medium was added to each well at day 4, and the whole medium was changed at day 7. We took pictures of the cell aggregation in a random selection after 10, 13, and 21 days of culture using an inverted microscope (Nikon Instrument, Melville, NY, USA) and a camera (D80, Nikon). 

### 4.2. Changes in Osmotic Pressure

Defined High-OP medium (High-OP: 450 ± 10 mOsm) was made with supplementally added sodium chloride. We added 4.6 g/L of sodium chloride to DMEM/Ham’s F-12 and sterilized it with a filter (0.45 µm, Nalgene™). The defined OP was confirmed with an osmometer (Osmet™, Precision, Natick, MA, USA) for each preparation. Regular medium measured 310 ± 10 mOsm, which was considered a low OP (Low-OP). 

### 4.3. Measurement of Gene Expression by Quantitative Polymerase Chain Reaction (qPCR)

We extracted total RNA from at least 48 spheroids harvested from 48 wells using Trizol™ (Life Technology), following the manufacturer’s instructions. Optical density and fluorescent intensity of the RNA was measured with a spectrophotometer (NanoDrop™, NanoDrop Technologies, Wilmington, DE, USA) and a fluorometer (Bioanalyzer, Agilent, Santa Clara, CA, USA), respectively. The sample (<500 ng) was mixed with TaqMan^®^ (Life Technology) for the specific gene and was followed by qPCR with an ABI Prism 7300 system (Applied Biosystems, Foster City, CA, USA). The genes of interest were chosen as follows: aggrecan core protein (*Agg*, Bt03212189_m1), collagen type Iα1 (*Col-1*, Bt_03225358_g1), collagen type IIα1 (*Col-2*, Bt_03251837_m1), matrix metalloproteinase-13 (*Mmp-13*, Bt_03214051_m1), and glyceraldehyde 3-phosphate dehydrogenase (*Gapdh*, Bt_03210919_g1). The expression of each gene was normalized to that of *Gapdh* and expressed as a relative quantity (RQ). The mean of the RQ from each gene was compared with various culture conditions versus the DZ at day 10, defined as RQ = 1.000. 

### 4.4. Histological Characteristics

We used at least 48 spheroids harvested from 48 wells for histological evaluation. To evaluate specific components of ECM in spheroids, immunohistochemical staining was conducted using antibodies against-KS, COL-1, COL-2, and MMP-13. 

We harvested spheroids at day 21, fixed them in 2% paraformaldehyde/0.1 M sodium cacodylic acid (pH 7.4), and then embedded them in 1.5% agarose gel to ease handling for further histological processing. The spheroids within the agarose gels were dehydrated in graded ethanol, replaced with xylene, and embedded in paraffin. The samples in paraffin were sectioned into 10-µm thick pieces, dewaxed with xylene, and then rehydrated with graded ethanol for immunohistochemical staining. The rehydrated sections were treated with 0.3% hydrogen peroxide in water for twenty minutes at room temperature. Then, the sections were blocked with 3% normal horse serum (Vector Laboratories, Burlingame, CA, USA) for twenty minutes in a humidified chamber. We followed the manufacturer’s instructions (Vector Laboratories) for further staining. Primary antibodies were selected to identify ECM components. The dilution of each antibody was 1:500 for mouse monoclonal anti-KS (Seikagaku America, East Falmouth, MA, USA), 1:150 for rabbit polyclonal anti-bovine COL-1 (Abcam, Cambridge, MA, USA), 1:50 for rabbit polyclonal anti-bovine COL-2 (Chemicon International, Temecula, CA, USA), and 1:150 for rabbit polyclonal anti-bovine MMP-13 (LifeSpan BioSciences, Seattle, WA, USA). Color was developed with 3,3-diaminobenzidenetetrahydrochloride-nickel kits, with or without nickel (Vector Laboratories). Counter-staining was conducted with Contrast Red™ (KPL, Gaithersburg, MD, USA) or hematoxylin (SIGMA-Aldrich, St. Louis, MO, USA). Immunologically positive components were identified by their black deposits with red nucleic staining, using Contrast Red, or by brown deposits with blue nucleic staining, using hematoxylin.

### 4.5. Data Analysis of Gene Expression Profiles Using a qPCR Assay

RQ was the index number used to compare to the defined sample expressed as 1.0. We chose the DZ at day 10 as the defined sample because this sample had the lowest expression of the typical phenotypic marker of chondrocytes, namely aggrecan, when compared with *Gapdh*. We compared the RQ of each zone to the RQ of DZ at day 10. We analyzed the RQ using one-way analysis of variance (ANOVA) to compare LL and LH at day 13, as well as LLL and LHH or LHL at day 21, after conducting a Smirnov–Grubbs test with four values obtained from four experiments. The RQ with *p* < 0.05 was considered statistically significant (GraphPad InStat ver. 3.00, San Diego, CA, USA).
